# Ruminal dysbiosis-induced mastitis: new insight into the pathogenesis of mastitis

**DOI:** 10.1186/s40104-025-01253-2

**Published:** 2025-10-27

**Authors:** Caijun Zhao, Xiaochun Sun, Naisheng Zhang, Xiaoyu Hu, Hongyan Li, Yunhe Fu

**Affiliations:** 1 Department of Urology, China-Japan Union Hospital of Jilin University, Changchun, 130033 China; 2 Department of Clinical Veterinary Medicine, College of Veterinary Medicine, Jilin University, Changchun, 130062 China; 3 Department of Gynecology, China-Japan Union Hospital of Jilin University, Changchun, Jilin 130033 China

**Keywords:** Bacterial translocation, Mastitis, Metabolic disorder, Pathogenesis, Ruminal microbiota

## Abstract

Mastitis is one of the most significant diseases affecting the development of the dairy industry and has traditionally been associated with pathogenic infections. However, emerging evidence highlights that ruminal microbial homeostasis also plays a crucial role in the pathogenesis of mastitis. Specifically, cows with mastitis exhibit reduced alpha diversity and altered microbial composition in the rumen. Inducing ruminal dysbiosis through a high-concentrate diet has been shown to trigger mastitis in cows, and transplantation of ruminal microbiota from mastitis-affected cows to recipient mice can induce mastitis in mice. Mechanistically, ruminal dysbiosis increases gastrointestinal inflammation and compromises the integrity of the gastrointestinal barrier, thereby facilitating the translocation of harmful bacterial components, metabolites, and pathobionts into the bloodstream. This disruption impairs blood-milk barrier function, leading to systemic inflammation and the development of mastitis. In this review, we summarize recent advances in understanding how ruminal dysbiosis induces mastitis and explore potential prevention and control strategies targeting the modulation of ruminal microbiota.

## Introduction

A healthy ruminal microbiota plays a pivotal role in maintaining normal lactation by supplying the precursor substances necessary for milk component synthesis, particularly volatile fatty acids (VFAs), such as acetate, propionate, and butyrate [1–4]. In the rumen, Firmicutes and Bacteroidetes dominate due to their cellulolytic and hemicellulolytic capabilities, with *Prevotella* being especially prominent [2, 5, 6]. These bacteria, along with ciliates, break down structural carbohydrates into pyruvate, which is subsequently converted into acetate, butyrate, and propionate through various carbohydrate-active enzymes (CAZymes) [7, 8]. However, several factors—including negative energy balance (NEB), ketosis, prolonged consumption of high-grain diets, and abrupt feed transitions during the perinatal period—can cause ruminal dysbiosis, thereby affecting the health of dairy cows and the development of diseases [1, 9, 10].

Mastitis is particularly significant in dairy cows and is commonly attributed to pathogenic infections in the mammary glands, e.g., *Staphylococcus aureus* (*S. aureus*) and *Escherichia coli* (*E. coli*) [11, 12]. However, an increasing number of studies have demonstrated that the proportion of milk samples with negative pathogen culture results in mastitis cases is increasing and that antibiotic treatment is ineffective for this type of mastitis [13, 14]. Moreover, the onset of mastitis is often linked to ruminal dysbiosis-related conditions, including SARA [10, 15], diarrhea [16], and ketosis [9]. Furthermore, research has shown that cows suffering from SARA exhibit elevated somatic cell counts (SCCs) in milk and display mastitis symptoms [10]. Ruminal microbiota transplantation (RMT) from cows to mice can induce mastitis in mice independently of mammary gland infection [15]. The role of ruminal dysbiosis in mastitis development has been further substantiated through cow-to-mouse RMT experiments involving cows with clinical mastitis [17]. Further studies have indicated that ruminal dysbiosis-associated inflammatory responses, particularly those triggered by lipopolysaccharide (LPS) and muramyl dipeptide (MDP), compromise both gastrointestinal barrier function and blood-milk barrier integrity, ultimately leading to systemic inflammation and the development of mastitis [17, 18]. The disruption of barrier integrity also facilitates the translocation of ruminal pathobionts into the mammary gland, such as *Stenotrophomonas* and Enterobacteriacea [10, 19, 20]. Additionally, ruminal dysbiosis can lead to metabolic disturbances, especially elevated levels of sialic acids and succinate, which further promote the expansion of pathobionts and exacerbate mastitis [15, 18, 21]. A reduction in beneficial metabolites, such as butyrate, deoxycholic acid (DCA), and 5-hydroxyindole acetic acid (5-HIAA), also plays a crucial role in regulating mastitis progression by enhancing barrier integrity and suppressing inflammatory responses [20, 22–24].

Herein, we comprehensively review the recent advances in ruminal dysbiosis-induced mastitis, with a focus on the pathogenesis involving systemic inflammatory responses, barrier dysfunction, subsequent pathobiont translocation, and metabolic disorders. Additionally, we elucidate the impact of ruminal dysbiosis on mammary gland infections and explore potential strategies targeting the rumen microbiota for the prevention and intervention of mastitis in dairy cows.

## Microbiota and metabolism in the healthy rumen

### Ruminal microbiota

The ruminal microbiota serves as a critical microecosystem enabling ruminants to digest various types of feed, particularly plant-derived resources, thereby supporting efficient milk production via microbial fermentation. A healthy rumen harbors a complex microbial community comprising bacteria, archaea, fungi, viruses, and protozoa, all of which play essential roles in rumen digestion and the overall health of dairy cows [1]. In this study, we focused primarily on reviewing the potential role of ruminal bacteria in regulating mammary health in dairy cows. Among the most abundant phyla in the rumen are Bacteroidetes, Firmicutes, and Proteobacteria (Table 1) [1, 4]. Unlike nonruminants, ruminants possess a unique capacity to utilize nutrients from plant fibers through microbial fermentation, with the resulting products accounting for the majority of milk precursors [2, 25]. Approximately 60%–70% of the metabolizable energy for cows is supplied by VFAs, which are produced through the degradation of plant cellulose and hemicellulose [26]. Firmicutes species, including *Ruminococcus flavefaciens* (*R. flavefaciens*), *Ruminococcus albus*, and *Butyrivibrio fibrisolvens* (*B. fibrisolvens*), and *Fibrobacter succinogenes* (*F. succinogenes*), which belong to Fibrobacteres, are the primary cellulolytic bacteria in the rumen [1, 27]. *Prevotella* (belonging to the Bacteroidetes), *B*. *fibrisolvens*, and *R*. *flavefaciens* are the predominant hemicellulose-degrading bacteria [1, 28]. These bacterial species are therefore considered the core members of the rumen bacterial microbiota [1, 2, 6, 27]. Additionally, *S. bovis*, *S*. *ruminantium*, and Proteobacteria such as *Ruminobacter amylophilus* and *Succinimonas amylolytica* play critical roles as starch-utilizing bacteria in the rumen [1]. Among these, *S*. *bovis* is a lactate producer that exhibits enhanced activity in high-starch diets, whereas *S*. *ruminantium* and *Megasphaera elsdenii* (*M. elsdenii*) consume lactate, converting it into propionic acid to prevent excessive lactate accumulation in the rumen [1, 29, 30]. Members of the Prevotellaceae family are also involved in protein and starch degradation, contributing to propionate production [31]. Given that propionate serves as the most important substrate for milk component synthesis, relatively high abundances of Prevotellaceae, *Succinimonas*, and *Selenomonas* have been observed in the rumens of high-producing dairy cows [4, 32, 33].

**Table 1:** Composition and function of rumen microbiota

Phylum	Family	Genus	Function or effects	References
Bacteroidetes	Prevotellaceae	*Prevotella*	Protein, carbohydrate degradation, VFA production (especially propionate), B and K_2_ vitamins synthesis, bile acid metabolism	[2, 4, 7, 34, 35]
	Rikenellaceae	*Rikenellaceae_RC9*	Not well been defined yet	
Firmicutes	Ruminococcaceae	*Ruminococcus*	Cellulose, hemicellulose degradation; B and K_2_ vitamin biosynthesis	[27, 34]
		*Clostridium IV*		
		*Saccharofermentans*		
		*Flavonifractor*		
		*Ruminococcaceae family*		
	Selenomonadaceae	*Selenomonas ruminantium*	Carbohydrate fermentation; Utilizing lactate to produce propionate	[4, 8]
	Veillonellaceae	*Megasphaera elsdenii*	Carbohydrate fermentation; Utilizing lactate to produce propionate	[30]
	Lactobacillaceae	*Lactobacillus*	Starch utilization	[36–38]
	Streptococcaceae	*Streptococcus*	Carbohydrate fermentation; Starch utilization; Lactate-producer	[29]
	Christensenellaceae	*Christensenellaceae_R-7 group*	Structural carbohydrates digestion; Producing acetate and butyrate	[39]
	Lachnospiraceae	*Butyrivibrio*	Cellulose, hemicellulose degradation, butyrate production	[27, 40]
		*Pseudobutyrivibrio*		
		*Coprococcus*		
		*Moryella*		
		*Lachnospiraceae_UCG-009*		
	Clostridiaceae	*Clostridium*	Cellulose and hemicellulose degradation; Organic acids, carbon dioxide, and hydrogen gas production	[1]
Proteobacteria	Succinivibrionaceae	*Succinivibrio*	Starch utilization and succinate production	[4, 40]
		*Succinimonas*		
Fibrobacteres		*Fibrobacter*	Cellulose and hemicellulose degradation	[2, 5, 6]
Archaea	Methanobacteriaceae	*Methanosphaera*	Methanol, CO_2_ and H_2_ utilization, methane production	[2, 5]
		*Methanobrevibacter*		

### Carbohydrate metabolism and VFA production

**Fig. 1:**
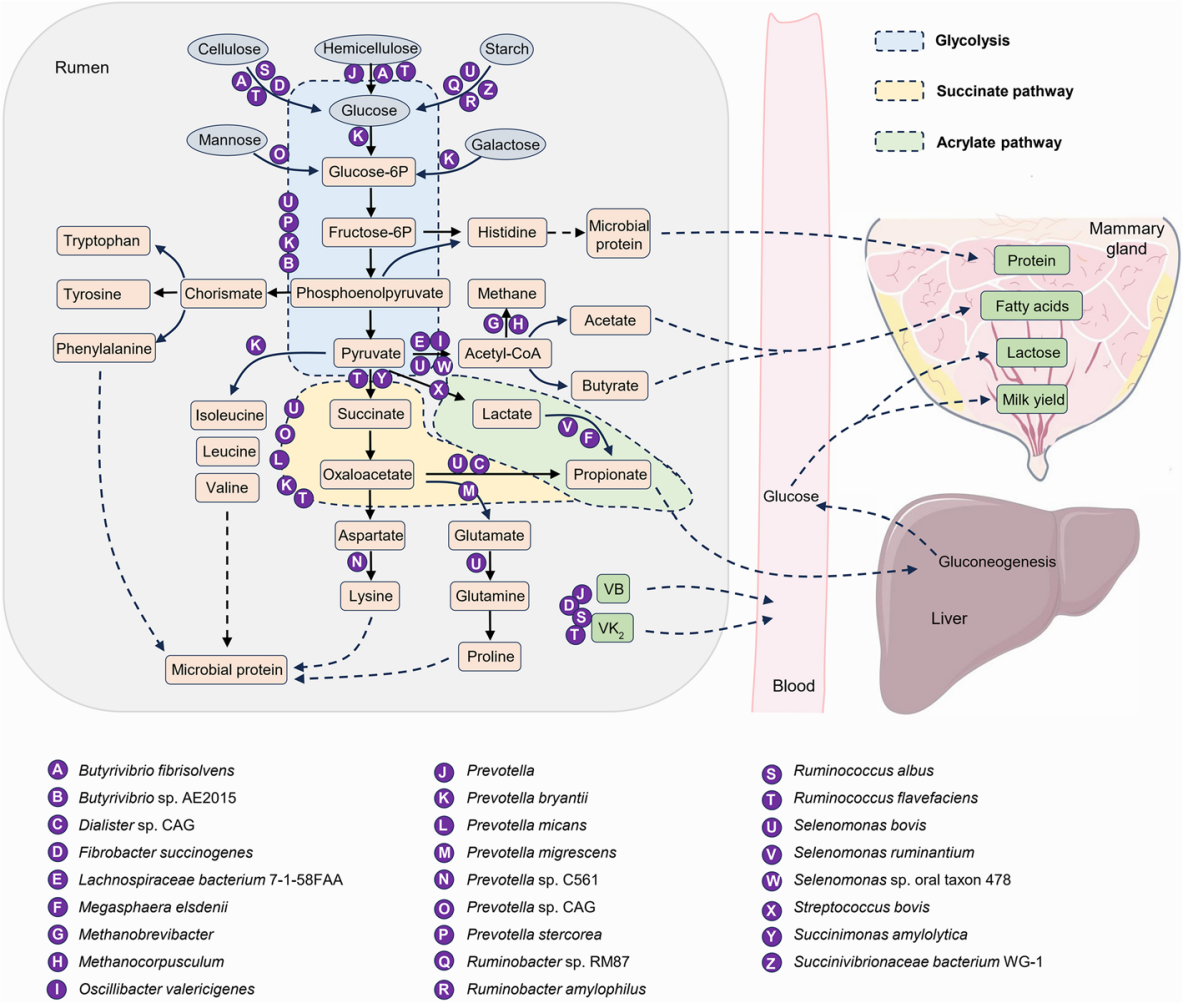
Rumen carbohydrate metabolism and VFA production. Dietary carbohydrates, including cellulose, hemicellulose, starch, mannose, and galactose, are metabolized into pyruvate via glycolysis by various ruminal bacteria such as *Prevotella*, *Fibrobacter* and *Ruminococcus* [1, 3, 8, 40]. Pyruvate is subsequently converted into acetate and butyrate through acetyl-CoA, contributing to the synthesis of mammary fatty acids. Furthermore, pyruvate is channeled into propionate production via the succinate and acrylate pathways, with propionate playing a critical role in hepatic gluconeogenesis for glucose production, which is closely linked to lactose synthesis and milk yield. Additionally, intermediates of glycolysis, such as fructose-6-phosphate, along with succinate, support the synthesis of amino acids and microbial proteins, thereby regulating milk protein production. The ruminal microbiota, particularly *Prevotella* and *Fibrobacter*, also facilitates the synthesis of B vitamins and vitamin K_2_ within the rumen

Cellulose, hemicellulose and starch are metabolized into glucose by the ruminal microbiota, and subsequently promoted pyruvate production by glycolysis. Pyruvate serves as a key intermediate in the production of VFAs, including acetate, butyrate, and propionate (Fig. 1). On the one hand, pyruvate is metabolized into acetyl-CoA, facilitating acetate and butyrate production. These VFAs are absorbed by ruminal epithelial cells into the bloodstream and transported to the mammary gland for fatty acid biosynthesis. Notably, *Papillibacter* and *Pseudobutyrivibrio* play critical roles in butyrate metabolism [40]. Additionally, acetyl-CoA can be used to produce methane by methanogenic bacteria such as *Methanobrevibacter* and *Methanocorpusculum* [2, 5], indicating that methanogenesis represents an energy-inefficient utilization pathway. On the other hand, pyruvate can be metabolized into propionate through lactate and succinate intermediates via the acrylate and succinate pathways, respectively. *R. flavefaciens* and *Succiniclasticum* contribute to propionate production via the succinate pathway [4, 40], whereas *S. ruminantium* is a well-known lactate-utilizing bacterium for propionate production via the acrylate pathway [4, 29]. Interestingly, cows with high feed efficiency exhibit increased relative abundances of propionate-producing microorganisms such as *Selenomonas* and *Succinivibrionaceae* [3, 8]. Propionate is absorbed into the bloodstream and transported to the liver, where it serves as a substrate for gluconeogenesis, leading to glucose production. This glucose subsequently travels via the bloodstream to the mammary gland for lactose synthesis. Consequently, a high level of rumen propionate is closely associated with increased milk yield [4]. Additionally, pyruvate can be metabolized into branched-chain amino acids (BCAAs), including valine, leucine, and isoleucine [40]. The intermediate oxaloacetate can be converted into glutamate and aspartate, thereby promoting the biosynthesis of amino acids such as proline and glutamine. Furthermore, intermediates such as fructose-6-phosphate and phosphoenolpyruvate can be transformed into histidine and aromatic amino acids, including tryptophan, tyrosine, and phenylalanine. These amino acids can be utilized by the ruminal microbiota for microbial protein synthesis, which enters the circulatory system and contributes to milk protein production.

### Vitamin biosynthesis

B and K_2_ vitamins are essential nutrients for host health and constitute critical components of milk that can be synthesized by the rumen microbiota. Studies based on microbial cultivation have demonstrated that certain specific rumen microbes, such as *Corynebacterium vitaeruminis* DSM 20294T [41], *F. succinogenes* [42], and *S. ruminantium* [43], can synthesize B vitamins. 16S rRNA sequencing has revealed that *Prevotella*, *Bacteroides*, and *Ruminococcus* are associated with B vitamin levels [44, 45]. Notably, the synthesis of B vitamins varies across different gastrointestinal regions. Biosynthesis pathways for riboflavin, pantothenate, biotin, folate, cobalamin, and menaquinone (K_2_ vitamin) are more prevalent in the stomach, whereas pathways for thiamine, niacin, and pyridoxine are more enriched in the large intestine [5, 34]. In the rumen, the B and K_2_ vitamin biosynthetic genes are predominantly assigned to the phyla Bacteroidetes and Firmicutes, with a relatively low proportion attributed to Proteobacteria [34]. Similarly, metagenomic data indicate that *Prevotella*, *Bacteroides*, *Clostridium*, *Ruminococcus*, *Methanobrevibacter*, *Fibrobacter*, and *Alistipes* harbor genes related to B and K_2_ vitamin biosynthesis, with *Prevotella* being the most prevalent taxon for vitamin biosynthesis in the rumen [5, 34].

## Rumen microbiota succession during the perinatal period

During the perinatal period, the adaptation of the rumen microbiota to varying nutritional strategies is critical for the transition from pregnancy to lactation. Rumen health is closely associated with dynamic changes in microbial composition and abundance, which evolve significantly from the prepartum to postpartum stages [1, 46]. The perinatal period in dairy cows typically spans 3 weeks before calving to 3 weeks after calving. Prior to calving, cows often experience a decrease in dry matter intake (DMI), which is particularly pronounced in primiparous cows. Given the substantial nutrient demands for prenatal fetal growth and the rapid increase in postnatal milk production, cows face an elevated requirement for nutrients such as protein. However, due to the persistently low DMI, cows enter a state of NEB. To compensate for this imbalance between energy intake and postpartum nutrient requirements, body tissues are mobilized to meet the demands of lactation. Although this process helps alleviate NEB, it may also compromise host health. For example, glycerol and free fatty acids derived from tissue mobilization often accumulate in the liver, imposing a metabolic burden [47]. Additionally, the increased levels of cytokines and reactive oxygen species (ROS) resulting from lipid and protein mobilization can trigger both local and systemic inflammatory responses [48, 49]. Moreover, NEB has been reported to reduce the sensitivity of the immune system, leading to immunosuppression in dairy cows [50, 51]. These characteristics may be responsible for the increased incidence of diseases, such as ketosis and mastitis, in dairy cows during the perinatal period [1, 52]. Indeed, long-term NEB can induce metabolic disorders, including ketosis, a disease characterized by elevated levels of β-hydroxybutyrate (BHBA), acetoacetate, and acetone in body fluids [53]. Cows with ketosis typically exhibit high levels of butyrate, sucrose, BHBA, and valerate, along with low concentrations of glucose and propionate [54]. Butyrate and valerate can be converted into BHBA, thereby exacerbating ketosis. This alteration in ruminal metabolic profiles is mediated by changes in the ruminal microbiota. Studies have shown that cows with ketosis present increased abundances of acetate- and butyrate-producing Christensenellaceae, which are positively associated with BHBA and negatively associated with propionate [55], respectively. Additionally, a greater abundance of lactate-producing *S. bovis* and lower abundances of lactate-utilizing *M. elsdenii* and *S. ruminantium* have been observed in cows with ketosis [29]. Both *M. elsdenii* and *S. ruminantium* can use carbohydrates or lactate to synthesize propionate [1, 4, 8]. Furthermore, increased relative abundances of Bacteroidota, *Christensenellaceae*_R-7, *Ruminococcus*, and *Thermomonas*, along with a reduced abundance of the propionate producer *Prevotella*, have been detected in dairy cows with subclinical ketosis [56].

In addition to NEB, perinatal changes in feed structure represent another critical factor influencing rumen microbiota dynamics. Typically, to meet the energy demands during the postpartum period and maximize milk production, a widely adopted feeding strategy involves transitioning from a fiber-rich diet to one with relatively lower fiber content and higher grain content. This dietary shift predictably reduces the relative abundances of cellulolytic and hemicellulolytic bacteria, such as Bacteroidaceae and Ruminococcaceae [46], while increasing the abundances of carbohydrate- and lactic acid-utilizing bacteria, including *S. bovis* and Christensenellaceae [39]. Notably, Proteobacteria becomes one of the dominant phyla in dairy cows fed a grain-based diet because of its ability to metabolize soluble carbohydrates [57, 58]. Prevotellaceae, which effectively produces propionate through polysaccharide and protein degradation and starch fermentation [31], shows an increase in relative abundance following the dietary transition in the postpartum period [46, 59, 60]. Transitioning to a high-grain diet may increase the rumen fermentation rate beyond the rumen's absorption and buffering capacities, leading to a decline in ruminal pH and the onset of SARA [61]. Persistent low pH disrupts the ruminal microbiota, promoting the proliferation of starch-digesting bacteria (e.g., *S. bovis*) and the lysis of Gram-negative bacteria (e.g., *F. succinogenes* and *R. flavefaciens*), thereby increasing the production of harmful microbial metabolites, including LPS, lactate, and histamine [36, 61, 62]. Furthermore, studies have demonstrated that the relative abundances of starch-utilizing bacteria, such as *Prevotella* and *Lactobacillus*, are elevated in the rumens of cows with SARA [36–38], along with those of pathogenic *E. coli* and *Clostridium perfringens* [63]. However, the relative abundance of lactate-utilizing *M. elsdenii* varies inconsistently across different studies [61, 63].

## Ruminal dysbiosis-induced mastitis and its potential pathogenesis

**Fig. 2:**
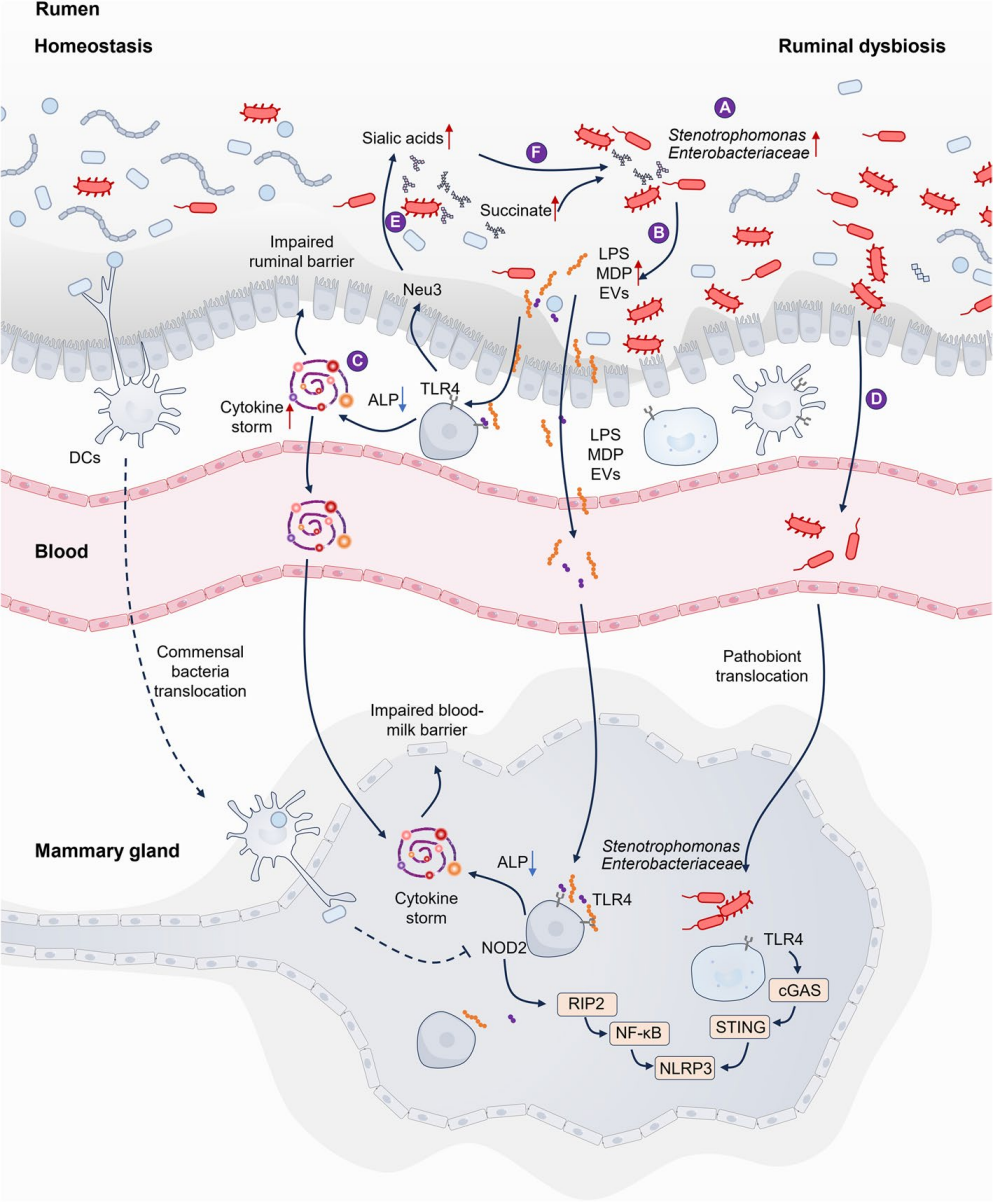
Potential mechanism underlying ruminal dysbiosis-induced mastitis. **A** Dietary changes and prolonged consumption of a high-grain diet can lead to ruminal acidosis, particularly promoting the expansion of pathobionts such as Stenotrophomonas and Enterobacteriaceae. This results in increased levels of LPS, MDP and EVs. **B** Increased levels of LPS and MDP enter the bloodstream and subsequently migrate to the mammary gland, where they activate TLR4 and NOD2, leading to the production of proinflammatory cytokines. The reduction in host ALP activity, which is mediated by TLR4-Neu3 signaling, further contributes to inflammatory responses. **C** Increased LPS levels also induce a cytokine storm in the rumen by impairing ALP activity through the TLR4-Neu3 signaling pathway, with proinflammatory cytokines potentially exacerbating mastitis directly. **D** Increased ruminal inflammation compromises ruminal barrier function, facilitating the translocation of pathobionts across the mucosal barrier. **E** Elevated Neu3 and bacterial sialidases contribute to the generation of free sialic acids, including Neu5Ac and Neu5Gc. **F** Sialic acids and succinate serve as carbon sources that promote the proliferation of ruminal pathobionts, thereby exacerbating dysbiosis-induced mastitis

Mastitis is typically categorized into subclinical and clinical forms based on SCC and udder-related clinical symptoms. During mastitis, lactation parameters such as milk yield and milk fat, protein, and lactose contents are significantly reduced, with the extent of reduction correlating with the severity of mastitis [64, 65]. In cows with mastitis, the diversity of the ruminal microbiota decreases, and the microbial community structure undergoes significantly changes (Table 2) [15, 17]. Specifically, the abundances of ruminal Proteobacteria, *Moraxella*, *Rikenellaceae*_RC9_gut_group, and *Saccharofermentans* increase in mastitis cows based on 16S rRNA sequencing [17]. Intriguingly, the abundances of *Moraxella* and Neisseriaceae in the rumen progressively increase as mastitis develops [64]. Furthermore, the abundance of *Prevotella*, a genus known for its role in propionate production in the rumen, is markedly reduced during mastitis [17, 64]. In addition to pathogen infection, ruminal dysbiosis can directly lead to mastitis. For example, cows fed a high-grain diet (70% grain and 30% forage) for 8 weeks developed mastitis, as indicated by the elevated SCC in milk; increased mammary gland injury; enhanced proinflammatory cytokine production; and ruminal dysbiosis characterized by an increased abundances of Proteobacteria, Moraxellaceae, and *Stenotrophomonas*, and reduced ruminal *Prevotella* abundance [10, 15]. Transplantation of this dysbiotic ruminal microbiota into mice induced mastitis in the recipients [15]. Consistently, ruminal RMT from cows with clinical mastitis also triggered mastitis in mice [17]. Interestingly, RMT primarily induces microbial alterations in the colon of mice [66]. Similarly, fecal microbiota transplantation (FMT) from cows with mastitis to mice caused mastitis without detectable pathogen infection [20, 67]. In a mouse model, gut dysbiosis induced by long-term antibiotic treatment also led to mastitis [22, 68]. Here, we further summarize recent advances and elucidate the molecular mechanisms underlying ruminal dysbiosis-induced mastitis, with a focus on systemic inflammation, disruption of the blood-milk barrier, bacterial translocation, and metabolic disturbances (Fig. 2).

**Table 2:** Changes of ruminal microbiota in cows with mastitis

Mastitis types	Treatment	Diagnostic criteria	Microbial changes	References
SARA-associated mastitis caused by a high-grain diet	Cows were fed a high-grain diet comprising 30% forage and 70% mixed concentrate for 8 weeks after 15 days adaptation	SCC > 15 × 10^5^ cell/mL	Proteobacteria, *Stenotrophomonas*, and *Succinivibrio* increased in the rumen; Firmicutes, Ruminococcaceae, and Rikenellaceae decreased	[10]
SARA-associated mastitis	Treatment with a high-grain diet (70% grain) for 8 weeks		Proteobacteria, *Moraxella*, *Pseudomonas*, *Paracoccus*, *Luteimonas* increased Prevotellaceae and *Candidatus_Saccharimonas* decreased	[15]
Clinical mastitis		SCC > 5 × 10^5^ cell/mL and macroscopical inflammatory changes	Proteobacteria, *Moraxella*, *Rikenellaceae*_RC9_gut_group, *Saccharofermentans*, Eubacterium_nodatum_group increased; Prevotellaceae and Bacteroidota decreased	[17]
Clinical mastitis		SCC > 3 × 10^6^ cell/mL, with obvious signs of inflammation in udders; positive California mastitis test (CMT) results	*Pseudobutyrivibrio*, *Gastranaerophilales*, *Moraxella*, and Neisseriaceae increased; *Prevotella_1, Lachnospiraceae*, *Mollicutes*_RF39, and *Bifidobacterium* decreased	[64]
Clinical mastitis		SCC > 1 × 10^6^ cell/mL	*unclassified_f_RF16*,* Paenibacillus*,* unclassified_c_Deltaproteobacteria*,* unclassified_f _BS11*, *unclassified_f _Succinivibrionaceae*,* Lysinibacillus*,* unclassified_f_Pirellulaceae*,* unclassified_o _GMD14H09* and *Sutterella* increased; unclassified_f _*Succinivibrionaceae*, *Rhodobacter*, *Comamonas*, *Enterococcus*, and unclassified_c_*Gammaproteobacteria* decreased	[65]
Clinical mastitis		SCC > 1 × 10^6^ cell/mL; Positive CMT results	*Prevotella 1*, Prevotellaceae *UCG001*,* Prevotellaceae UCG003*,* Fibrobacter* increased; *Ruminococcaceae* UCG 014, *Eubacterium coprostanoligenes* group, *Eubacterium ruminantium *group, *Ruminococcus* 1, *Syntrophococcus*, *Dialister*, *Pseudobutyrivibrio*, *Desulfovibrio*, *Lachnoclostridium* 12, *Ruminococcaceae* UCG 007, *Peptostreptococcus*, *Mitsuokella* decreased	[69]
Subclinical mastitis		6 × 10^5^ < SCC < 1 × 10^6^ cells/mL; no obvious clinical symptoms of mastitis in the udder; CMT results were weakly positive	*Ruminiclostridium*_9, *Bacteroidales*_UCG-001, and *Enterorhabdus* increased; *Prevotella_1, *Lachnospiraceae, *Mollicutes*_RF39, and *Bifidobacterium* decreased	[64]
Ketosis-associated mastitis			*Christensenellaceae*_R-7, *Chryseobacterium*, *Rikenellaceae*_RC9_gut_group increased; *Prevotella* decreased	[9]

### Systemic inflammation

In cows with mastitis, elevated levels of serum proinflammatory cytokines and LPS were observed [17, 20]. Notably, altered liver enzyme profiles, such as increased serum alanine aminotransferase and aspartate aminotransferase levels alongside reduced alkaline phosphatase (ALP) activity, were observed in mastitis-affected cows. These changes are associated with elevated ruminal *Moraxella* levels [17], suggesting that ruminal dysbiosis may contribute to liver injury and systemic inflammation. During ruminal dysbiosis, particularly when induced by SARA, increased ruminal Proteobacteria abundances promote the release of LPS. Although low-level LPS exposure does not immediately trigger a significant inflammatory response due to the detoxifying action of host anti-inflammatory enzymes such as ALP, which dephosphorylates LPS [17], repeated LPS exposure progressively impairs host defense mechanisms by inducing endogenous neuraminidase (Neu) activity [17, 70]. Specifically, LPS activates Toll-like receptor (TLR)4, leading to increased Neu3 levels and accelerating the desialylation-mediated molecular aging and clearance of the intestinal ALP [70, 71]. Consequently, recurrent LPS stimulation induces TLR4-dependent inflammation in both the gastrointestinal tract and bloodstream [70, 71]. ALP is produced by various tissues, including the colon, liver, and mammary gland. In the mammary gland, repeated LPS exposure also impairs ALP activity, thereby contributing to mastitis through activation of the TLR4-cGAS-STING-NF-κB/NLRP3 pathway [17]. In addition to LPS, ruminal dysbiosis may promote the release of peptidoglycans, particularly MDP, which induces the activation of host nucleotide-binding oligomerization domain-containing protein 2 (NOD2) and stimulates proinflammatory cytokine production via the RIP2-NLRP3 pathway [18]. Notably, immune cells such as macrophages, when chronically exposed to LPS, can be reactivated by MDP stimulation, leading to enhanced proinflammatory responses [72]. These findings suggest that ruminal dysbiosis-derived LPS and MDP may synergistically contribute to mastitis development, although further studies needed to confirm this hypothesis. Additionally, acute gastrointestinal inflammation directly elevates the levels of proinflammatory cytokines, such as TNF-α and IL-1β, in the bloodstream, which coincides with exacerbated symptoms of mastitis. However, systemic inflammation and mastitis were alleviated following the resolution of intestinal inflammation [73], indicating that the cytokine storm induced by ruminal dysbiosis plays a critical role in mastitis progression.

NEB during the perinatal period can lead to metabolic and immunological disturbances in dairy cows, triggering systemic inflammatory responses [74]. Furthermore, NEB may impair leukocyte function and increase the susceptibility to mastitis [75]. Specifically, NEB and elevated serum BHBA levels have been associated with reduced leukocyte activity, thereby increasing the risk of intramammary infection in cows [76]. In addition, high serum BHBA concentrations can impair the ability of leukocytes to migrate to the infected mammary gland [77]. These findings are further supported by evidence showing that cows with prepartum ketosis are more prone to developing mastitis [77]. However, it remains unclear whether the impaired immunity and dysregulated inflammatory responses caused by NEB and ketosis are modulated by the ruminal microbiota. Similar to NEB, hypocalcemia is also a key factor influencing immune function and promoting the development of mastitis [78, 79]. Cows affected by hypocalcemia exhibit decreased plasma insulin concentrations, elevated glucose levels, and reduced neutrophil phagocytic and oxidative burst capacities following pathogen exposure [80]. Low Ca^2+^ levels also interfere with glycolysis, thereby impairing neutrophil adhesion, phagocytosis, and extracellular trap formation [81, 82].

### Compromised barrier function

Under physiological conditions, a tightly regulated gastrointestinal barrier consisting of immune cells, epithelial cells, and a mucus layer maintains the anaerobic environment in the gastrointestinal tract while preventing direct contact between commensal bacteria and host tissues. Epithelial barriers are established through intercellular tight junctions (TJs), which primarily consist of transmembrane proteins such as claudins, occludin, and tricellulin and are directly responsible for maintaining barrier integrity [83]. The *zonula adherens* indirectly supports barrier function by promoting TJ formation [83]. Disruption or even minor impairment of barrier function can trigger severe inflammatory responses [84]. In cows with mastitis, decreased levels of occludin, claudin-3, and zonula occludens (ZO)-1 have been observed in the rumen [10, 17]. Similarly, in mice with RMT- or FMT-induced mastitis, comparable findings have been reported [17, 20]. Furthermore, microbial dysbiosis during mastitis reduces mucin-2 expression, which coincides with a decrease in goblet cell counts and suggests compromised mucosal barrier function [15, 17, 20].

Another pathophysiological mechanism underlying ruminal dysbiosis-induced mastitis is the disruption of the blood-milk barrier, which is formed by mammary epithelial cells that separate the mammary acinus from the circulatory system [85]. Under homeostatic conditions, the integrity of the blood-milk barrier ensures normal exchange of substances between the blood and mammary acinus while preventing excessive infiltration of inflammatory mediators and pathogens [85]. However, increased permeability of the blood-milk barrier in response to various ruminal dysbiosis-associated factors, such as inflammatory cytokines, harmful metabolites, and pathobionts, can promote the development of mastitis [86]. For example, LPS, an inflammatory mediator derived from ruminal dysbiosis, can downregulate the expression of TJs, thereby compromising blood-milk barrier integrity during mastitis [87]. In cows with ruminal dysbiosis-induced mastitis and in recipient mice following RMT, elevated serum LPS levels and reduced mammary TJ expression have also been observed [17]. Proinflammatory cytokines such as TNF-α can disrupt TJs through multiple mechanisms, including IRF1- and caspase-3-mediated pyroptosis and NF-κB activation [88, 89]. Another key cytokine, IL-1β, increases epithelial TJ permeability by upregulating MIR200C-3p, which leads to occludin mRNA degradation [90]. Additionally, IL-6 compromises epithelial barrier integrity by modulating STAT3 signaling [91]. Consistently, increased systemic inflammation and impaired blood-milk barrier integrity have been confirmed in cows with ruminal dysbiosis-induced mastitis [10, 15, 17]. Moreover, microbial metabolites also play significant roles in regulating blood-milk barrier integrity. For instance, butyrate protects against LPS-induced disruption of the blood-milk barrier by activating HDAC3 in mice [92]. Activation of the aryl hydrocarbon receptor (AhR) by microbial tryptophan metabolites can also enhance epithelial TJ integrity by suppressing NF-κB-mediated inflammatory responses and activating the Nrf2 pathway [93, 94]. Collectively, these findings indicate that microbial dysbiosis-mediated disruption of blood-milk barrier integrity serves as a critical regulatory mechanism in the pathogenesis of mastitis.

### Alteration of the milk microbiome and bacterial translocation

Recent studies have confirmed that the mammary gland harbors its own microbial ecosystem, consisting of both pathogenic bacteria capable of causing mastitis and milk spoilage, such as *Staphylococcus*, *Pseudomonas*, and *Streptococcus*, as well as probiotic strains including *Lactococcus*, *Lactobacillus*, *Pediococcus*, *Leuconostoc*, and *Enterococcus* species were existed [95, 96]. Kim et al. [97] demonstrated that Proteobacteria, Firmicutes, Actinobacteria, and Bacteroidetes are the dominant microbial phyla, with Proteobacteria being the most diverse and abundant group in bovine milk in Korea. Another study reported that Firmicutes (40.8%) was the most prevalent phylum, followed by Proteobacteria (39.0%), Actinobacteria (9.40%), and Bacteroidetes (7.47%) [98]. At the genus level, *Pseudomonas* (19.6%), *Bacillus* (13.8%), *Lactococcus* (11.7%), and *Acinetobacter* (10.2%) were identified as the predominant genera in milk [98]. Although the microbial composition of bovine milk varies across studies, most reports indicate that Proteobacteria, including *Acinetobacter*, *Pseudomonas*, *Escherichia*, *Vibrio*, *Erwinia*, and *Pantoea*, constitute the major bacterial group, accounting for up to 90% of the milk microbiota. The characteristics of milk-associated microorganisms have been comprehensively reviewed elsewhere [95]. During mastitis, significant alterations occur in the milk microbiota. For instance, in milk samples from cows with clinical mastitis, the relative abundances of Firmicutes and Actinobacteria were found to decrease, while those of Tenericutes and Fusobacteria were elevated [99]. Wang et al. [100] also reported elevated levels of *Staphylococcus*, *Streptococcus*, *E. coli*, *Klebsiella*, *Pseudomonas* in the milk of mastitis cows. Moreover, using 16S rRNA sequencing, Zhong et al. [65] observed that cows with high somatic cell counts (> 50 × 10^4^ cells/mL) exhibited reduced milk yield, altered milk composition, and lower ruminal volatile fatty acid concentrations, along with decreased abundance of Succinivibrionaceae. Similarly, Zhang et al. [101] found that mastitis cows had lower lactose and fat content in milk, which correlated with reduced ruminal SCFA levels and increased abundances of Bacteroidetes, Firmicutes, Lachnospiraceae, *Prevotella*, and Rumiclostridium in the rumen. In contrast, cows supplemented with rumen-native microbes such as *C. beijerinckii*, *P. kudriavzevii*, *Ruminococcus bovis*, and *B. fibrisolvens* showed improvements in milk yield, as well as fat and protein contents [102]. These findings highlight the crucial role of the ruminal microbiota in modulating the milk microbiome and influencing milk composition.

Interestingly, the origins of bovine milk microbiota are believed to be derived from both the surrounding environment and endogenous translocation [95]. For example, *Bifidobacterium animalis* subsp. *lactis* Probio-M8 translocates to the mammary glands via entero-mammary routes during lactation [103]. Similarly, *Lacticaseibacillus rhamnosus* Probio-M9 can translocate into the mammary glands during both lactation and mastitis [104]. Additionally, the oral administration of *Lactococcus lactis* MG1614 and *Lactobacillus salivarius* PS2 results in their translocation into milk and mammary glands during pregnancy [105]. Moreover, supplementation with *Lactobacillus fermentum* CECT5716 in pregnant and lactating rats affects mammary milk composition through direct translocation [106]. Unlike the translocation of pathobionts, which depends on impairment of the gut barrier, the translocation of commensal bacteria does not compromise the integrity of the intestinal epithelial barrier. This suggests the existence of a potential entero-mammary axis under physiological conditions [95]. Studies have shown that the surface antigens of commensal microbes can be recognized and engulfed by monocytes, including dendritic cells and macrophages, which are widely distributed in the gut. These monocytes, after engulfing commensal microbes, traverse the TJs of intestinal epithelial cells and reach the mesenteric lymph nodes. These monocytes can subsequently be directly transported to the mammary glands via the blood-lymphatic system, through which they eventually reach maternal milk [95, 107]. Supporting evidence for this hypothesis includes findings that oral administration of *Lactobacillus* resulted in the detection of the same strain in Peyer’s patch cells in mice [105]. Another study demonstrated the simultaneous presence of *Ruminococcus*, *Bifidobacterium*, and Peptostreptococcaceae in feces, milk, and blood leukocytes in dairy cows, suggesting that circulating white blood cells may play a role in the translocation of microbes across the entero-mammary axis. However, further studies are needed to confirm these findings [108].

Disruption of the gastrointestinal barrier also promotes the proliferation of gut pathobionts and their translocation to distant organs and tissues. For example, *Enterococcus gallinarum* can compromise the gut barrier and translocate into liver and systemic tissues, thereby contributing to autoimmunity [109]. Similarly, *Klebsiella pneumoniae* disrupts the epithelial barrier, initiating bacterial translocation and triggering liver inflammatory responses through the activation of T helper 17 cells [110]. Jiang et al. [99] demonstrated that *Sphingomonas* and *Stenotrophomonas* existed increased abundances in cows with mastitis, while *Lactococcus* was depleted. Another study also showed that, consistent with the increased relative abundance of *Stenotrophomonas* in the rumens of mastitis-affected cows [10], elevated levels of *Stenotrophomonas* in milk were detected during mastitis [10], suggesting potential bacterial translocation across the entero-mammary axis. Indeed, the administration of ruminal *Stenotrophomonas* to mice induces mastitis and impairs the blood-milk barrier [19]. Intriguingly, oral gavage of GFP-labeled *Stenotrophomonas* results in increased GFP signals in the mammary glands, confirming the translocation of *Stenotrophomonas* from the gut to the mammary glands [19]. Mechanistically, *Stenotrophomonas* activates the calcium-ROS-AMPK-mTOR signaling pathway, leading to mastitis and barrier dysfunction via autophagy induction [19].

In cows with mastitis, increased Enterobacteriaceae abundances were detected in both feces and milk. FMT from mastitis-affected cows to mice induced mastitis; however, this effect was reversed by treatment with ciprofloxacin, an antibiotic that targets Enterobacteriaceae [20]. Further results demonstrated that pathogenic *E. coli* can translocate into the mammary gland during gut dysbiosis-induced mastitis [20]. In mice with acute gut inflammation-associated mastitis, enrichment of *Escherichia*/*Shigella*, *Clostridium*, and *Streptococcus* was observed in both the gut and mammary glands [73]. *Clostridium* has also been identified as a pathobiont contributing to the development of preeclampsia via bacterial translocation [111]. Interestingly, many pathogens associated with metritis in cows, such as *Bacteroides*, *Porphyromonas*, and *Fusobacterium*, can be detected in both feces and blood during metritis, suggesting that blood serves as a route for the transmission of uterine pathogens from the gut to the uterus in cows [112]. Another study revealed that cows with mastitis presented increased abundances of *Klebsiella oxytoca* and *Nocardia pseudobrasiliensis* in both feces and milk and that microbiota transplantation from either source induced mastitis in mice [113], supporting the potential for bacterial translocation across the entero-mammary axis and its role in mastitis pathogenesis.

### Metabolic disorders

Alterations in metabolism represent the most prevalent mechanism for interactions between the gut microbiota and the host. Studies have demonstrated that cows with mastitis exhibit distinct metabolic profiles in the rumen. In SARA-associated mastitis, sialic acids, including N-glycolylneuraminic acid (Neu5Gc) and N-acetylneuraminic acid (Neu5Ac), were identified as the most differentially enriched metabolites in mastitis cows. This finding correlates with the enrichment of sialic acid-utilizing opportunistic pathogenic Moraxellaceae and the depletion of the sialic acid-utilizing commensal Prevotellaceae [15]. Sialic acids are nine-carbon backbone monosaccharides that are abundantly expressed on all mucosal surfaces but are present at relatively low levels in the gastrointestinal tract under normal conditions [114, 115]. These free sialic acids serve as nutrient sources for commensal bacteria, such as *Lactobacillus* [15]. However, microbial dysbiosis or mucosal inflammation enhances the release of free sialic acids. For example, antibiotic-induced gut dysbiosis increases fecal sialic acid levels [115]. In Gram-negative pathogen infections and LPS-induced inflammation, TLR4 activation upregulates Neu expression, thereby promoting sialic acid production [70, 71]. In cows with SARA-associated mastitis, increased ruminal sialidase activity was observed [15]. Consistent findings were reported in RMT mice. Notably, the treatment of these RMT mice with the Neu inhibitor zanamivir alleviated ruminal dysbiosis-induced mastitis. Mechanistically, under dysbiotic conditions, sialic acids serve as a carbon source for pathobionts [15]. Specifically, Neu5Ac predominantly promotes the growth of Enterobacteriaceae and enhances the expression of virulence factors such as *ler* and *tir*, exacerbating mastitis [15]. Conversely, Neu5Gc primarily drives the expansion of *Enterococcus*, increasing MDP release and inducing mastitis via the NOD2-RIP2-NF-κB pathway [18]. Another metabolite contributing to the development of mastitis is succinate, which is found to be elevated in the rumen during mastitis [15]. Succinate serves as a substrate for bacterial metabolism, particularly for Prevotellaceae, which produces propionate. The reduction in Prevotellaceae abundance during mastitis may account for the increased levels of succinate [15, 99]. Like sialic acids, succinate can promote the expansion of pathobionts, such as *Salmonella enterica serovar *Typhimurium and *Clostridium difficile* [116]. Additionally, elevated succinate exacerbates gut dysbiosis and stimulates the production of microbial extracellular vehicles containing LPS, thereby aggravating mastitis through TLR4 activation [18]. Cows with mastitis also exhibit increased metabolic pathways associated with valine, leucine, and isoleucine biosynthesis [67, 117]. Although increased isoleucine and valine have been shown to impair hepatic insulin sensitivity and ketogenesis, potentially facilitating obesity-associated metabolic syndrome [118, 119] their roles in the pathogenesis of microbial dysbiosis-induced mastitis remain unclear.

In addition to increased metabolites directly exacerbating mastitis, the depletion of beneficial metabolites also plays a critical role in this pathological process. For example, cows with mastitis exhibit reduced abundances of *Roseburia* and butyrate levels in the feces, as confirmed in FMT mouse studies [20]. Treating FMT-induced mastitis mice with *Roseburia* and butyrate not only alleviates mastitis symptoms but also restricts the translocation of pathogenic *E. coli* from the gut to the mammary glands [20]. Mechanistically, butyrate activates epithelial PPARγ, thereby reducing iNOS production and maintaining an anaerobic environment in the gut to limit Enterobacteriaceae expansion [120]. Furthermore, butyrate inhibits *E. coli*-induced inflammation by suppressing the activation of the TLR4-cGAS-STING-NF-κB/NLRP3 signaling pathway and restores the integrity of the blood-milk barrier [20]. Dysbiosis in the rumen also disrupts retinoic acid metabolism; administration of retinoic acid mitigates mastitis induced by recurrent low-grade endotoxemia via LPS stimulation in mice [121]. Additionally, vagotomy-induced mastitis in mice is associated with altered gut microbiota and microbial tryptophan metabolism, leading to decreased fecal levels of 5-hydroxyindole acetic acid (5-HIAA) [23]. Administration of 5-HIAA alleviates mastitis by inhibiting NF-κB activation through aryl hydrocarbon receptor (AhR) signaling [23]. Consistent with these findings, reduced ruminal 5-HIAA levels have been observed in cows with mastitis [15]. Moreover, several metabolic pathways linked to anti-inflammatory effects, such as lysine biosynthesis and vitamin B-related pathways (e.g., lipoic acid, folate, and thiamine metabolism), are depleted in cows with mastitis [67], although their specific roles in regulating mastitis require further investigation.

Other metabolites and bioactive small molecules, such as insulin, cortisol, and adipokines (e.g., leptin and adiponectin), have also been associated with the development of mastitis. For example, increased insulin resistance has been observed in LPS-induced mastitis [122]. Insulin-like growth factor 1 (IGF-1) and its receptor have been identified as indicators of ketosis and are linked to an increased susceptibility to mastitis in dairy cows [123, 124]. Studies have shown that disturbances in gut microbiota composition and their metabolic activities, such as reduced production of secondary bile acids and indole derivatives, can suppress glucagon-like peptide-1 (GLP-1) secretion, thereby contributing to elevated insulin resistance [125, 126]. Moreover, elevated cortisol concentrations were detected in cows with LPS-induced mastitis [127]. Interestingly, reduced mammary adiponectin levels were observed in cows with clinical mastitis, which were associated with peroxisome proliferator-activated receptor (PPAR) and adipocytokine signaling pathways [128]. Alterations in the gut microbiota can influence adiponectin expression, thereby modulating obesity and metabolic profiles [129]. Notably, although these biomarkers have been implicated in mastitis, their specific regulatory roles and underlying mechanisms in ruminal dysbiosis-induced mastitis remain to be fully elucidated.

## Impact of ruminal dysbiosis on pathogen infection in the mammary gland

In addition to inducing mastitis, ruminal dysbiosis may also exacerbate pathogen infection-induced mastitis. For example, gut dysbiosis caused by long-term antibiotic treatment aggravates *S. aureus* and *E. coli*-induced mastitis in mice [22, 68]. Specifically, antibiotic-induced gut dysbiosis depletes commensal bacteria such as *Clostridium* and *Lactobacillus reuteri* and impairs the production of SCFAs and indole derivatives, which limit pathogen-induced mastitis by inhibiting HDAC3 and activating AhR, respectively [22, 68]. Reduced levels of butyrate and the AhR ligand 5-HIAA are also observed in the feces or rumens of cows with mastitis [15, 20], suggesting that gastrointestinal dysbiosis may exacerbate bacterial infections in cows. Furthermore, alterations in secondary bile acid metabolism mediated by microbial activity are also implicated in mammary pathogen infections. Cows with mastitis exhibit reduced levels of cholic acid (CA) and deoxycholic acid (DCA) in milk, and DCA, but not CA, alleviates *S. aureus*-induced mastitis in mice [24]. Depletion of commensal bacteria capable of producing secondary bile acids by vancomycin also exacerbates *S. aureus*-induced mastitis. Mechanistically, DCA mitigates *S. aureus*-induced damage to the blood-milk barrier and inflammatory responses by inhibiting NF-κB and NLRP3 signaling through activation of the TGR5-cAMP-PKA pathways [24]. Notably, although the bile acid-metabolizing microbiome responsible for producing secondary bile acids predominantly resides in the large intestine [130], such as *Alistipes*, emerging evidence suggests that ruminal microbes, including Lachnospiraceae, Bacteroidaceae, and Acidaminococcaceae, particularly *Prevotella*, are also involved in bile acid metabolism [35]. Supplementing cows with cholic acid can increase ruminal SCFA levels while concurrently increasing the ruminal pH [35]. Conversely, a high-grain diet fed to cows increases cholic acid levels in the intestine, which correlates with an enhanced inflammatory response [130]. Other metabolites reduced during mastitis include hexadecanamide and phytosphingosine, both of which are present at relatively low concentrations in the rumens and milk of mastitis-affected cows [131, 132]. Treatment of mice with hexadecanamide and phytosphingosine alleviates *S. aureus*-induced mastitis by regulating the PPARα-SIRT1-NF-κB and NLRP3 pathways, respectively [131, 132]. Additionally, chronic inflammation induced by recurrent LPS stimulation exacerbates *E. coli*-induced mastitis in mice by impairing ALP activity [15].

## Targeting the ruminal microbiota for mastitis intervention

### Ruminal microbiota transplantation

Considering the critical role of the gut microbiota in disease development, modulating the microbiome via FMT is regarded as a promising strategy for intervention in diseases associated with gut dysbiosis. For example, FMT from healthy donors alleviated calf diarrhea and improved growth performance by reshaping the gut microbiota, particularly through an increase in Porphyromonadaceae and a reduction in the fecal amino acid concentration [133]. Furthermore, RMT enhanced feed efficiency in yaks by remodeling the ruminal microbiota, characterized by increased abundances of *Prevotella* and *Rikenellaceae*_RC9_gut_group [134]. Kong et al. [135] demonstrated that RMT could accelerate the transition process of the ruminal microbiota in postpartum dairy cows without significantly affecting DMI or feed efficiency [136]. Additionally, studies have shown that RMT can alleviate SARA and improve rumen health. For example, in a sheep model, RMT induced dynamic changes in rumen fermentation, increasing the concentrations of total VFAs, acetate, propionate, and butyrate while decreasing lactate and LPS levels in the rumen [136]. The administration of RMT increased the relative abundances of Bacteroidales, Prevotellaceae, and Ruminococcaceae, mitigated the damage to the rumen epithelium caused by acute rumen acidosis, and increased the length of the rumen papillae [136]. Moreover, RMT facilitated the restoration of rumen bacterial homeostasis and rumen fermentation in cows suffering from SARA without altering the core microbiome [137]. RMT also promoted the recovery of the rumen epithelial morphological structure but did not significantly enhance its functional recovery in SARA cows [138]. Interestingly, despite no significant changes in alpha diversity or the relative abundances of dominant genera such as *Ruminococcaceae* UCG-005 and *Eubacterium coprostanoligenes*, RMT altered the relative abundances of *Eubacterium oxidoreducens*, *Anaerorhabdus furcosa*, *Bacillus*, and *Selenomonas* in the feces of cows, which was consistent with changes in serum amino acid metabolism, bile acid metabolism, and fatty acid metabolism [139]. Given the role of SARA-associated dysbiosis in contributing to the development of mastitis, RMT may serve as a potential strategy for mastitis intervention, although this requires confirmation in future studies. Notably, RMT from adult ewes to preweaning lambs had adverse effects on the growth performance of weaned lambs by impairing gastrointestinal integrity and immunity and reducing feed intake and digestibility [140]. Another study revealed that the therapeutic effect of FMT for calf diarrhea was influenced by donor and recipient selection [141], indicating that the establishment of optimal donor-recipient selection criteria and standardized RMT procedures are critical prerequisites for the effective prevention and treatment of diseases using RMT.

### Probiotics

In addition to microbiota transplantation, the supplementation of probiotics may represent a direct approach for treating mastitis. For example, the administration of *Clostridium* species that produce SCFAs in mice alleviated *S. aureus*-induced mastitis [22]. Treatment with *Clostridium scindens*, a producer of secondary bile acids, also ameliorated *S. aureus*-induced mastitis by activating TGR5 through increased levels of DCA [24]. *Lactobacillus reuteri* (*L. reuteri*) treatment attenuated *E. coli*-induced mastitis by promoting tryptophan metabolism and activating AhR, leading to reduced NF-κB activation and improved barrier function in mice [68]. Additionally, *L. reuteri* alleviated gut dysbiosis-associated mastitis by competing with Enterobacteriaceae for sialic acid [15]. Mice treated with *Lactobacillus casei* exhibited improved mastitis symptoms and restored gut microbiota balance in cases of gut dysbiosis-induced mastitis [67]. *Roseburia*, an SCFA-producer depleted in cows with mastitis, improved mastitis outcomes by enhancing the blood-milk barrier integrity and limiting the translocation of Enterobacteriaceae across the entero-mammary axis in mice [20]. *Lacticaseibacillus rhamnosus* Probio-M9, a probiotic derived from milk, alleviates mastitis and enhances antibiotic efficacy [104]. Furthermore, *Bacillus amyloliquefaciens-*9 has been shown to reduce the SCC and modify the fecal microbiota in lactating goats [142]. The commensal *Bacillus subtilis* isolated from cow milk inhibited the biofilm formation of *S. aureus* and alleviated mastitis in mice [143]. In dairy cows, treatment with *Lactobacillus casei* Zhang and *Lactobacillus plantarum* P-8 significantly increased milk production, improved milk components (e.g., elevated lactoferrin and lysozyme levels), and decreased the SCC in milk [144]. These probiotics also increased the relative abundances of rumen fermentative bacteria, such as *Bacteroides*, *Roseburia*, *Ruminococcus*, *Clostridium*, *Coprococcus*, and *Dorea* [144]. Interestingly, a *Lactobacillus*-based disinfectant not only significantly reduced SCC but also improved bacterial communities in milk expressed from cow teats with subclinical mastitis [145].

### Prebiotics and dietary components

Dietary fiber serves as the primary substrate for the production of SCFAs by the commensal microbiota. Mice fed a fiber-enriched diet (e.g., inulin) presented increased relative abundances of SCFA-producing bacteria and elevated fecal SCFA levels, which alleviated *S. aureus*-induced mastitis by activating HDAC3-mediated antimicrobial programs in macrophages [146]. In cows with subclinical mastitis, dietary supplementation with inulin reduced the SCC in milk and decreased the concentrations of proinflammatory cytokines, including IL-6, IL-8, and TNF-α, while improving oxidative stress in the mammary glands [147]. Inulin treatment also increased the concentrations of propionate, butyrate, and lactate, while reducing NH3-N levels in the rumen, which was consistent with increased abundances of SCFA-producing bacteria such as *Prevotella* and *Butyrivibrio* in the rumen [147]. Dietary supplementation with citrus flavonoids increased the relative abundances of *Bacteroides*, *Bifidobacterium*, *Alistipes*, and *Akkermansia* in feces and regulated sphingolipid metabolism (e.g., reduced serum ceramide and sphingomyelin levels), leading to decreased levels of LPS and proinflammatory cytokines IL-6 and TNF-α in SARA cows [148]. Supplementing *Ampelopsis grossedentata* flavonoids increased ruminal alpha diversity and the abundance of *Prevotella_1* levels, which was associated with improved metabolic profiles [149]. Citrus flavonoid administration also alleviated mastitis and improved the integrity of the blood-milk barrier in mice [150, 151]. Feeding citrus flavonoids to dairy cows increased the abundances of *Ruminococcus*, *Clostridium*, and *Butyrivibrio*, while reducing the abundances of methanogens such as *Methanobacterium* and *Methanosarcina*, resulting in dose-dependent increases in the ruminal concentrations of total VFAs, acetate, propionate, butyrate, and microbial crude protein [152]. Consistently, treatment with citrus flavonoids increased milk production and lactose content in milk while linearly reducing the SCC, which was associated with decreased ruminal LPS levels and improved host antioxidant capacity [152]. Additionally, citrus flavonoids increased the total VFAs in feces and promoted the abundance of beneficial gut microbes, including fecal *Bifidobacterium*, *Clostridium coccoides-Eubacterium rectale* group, and *Faecalibacterium prausnitzii*, thereby alleviating systemic inflammation [153]. Notably, Zhong et al. found that ruminal *Erysipelotrichaceae* UCG 004 was correlated with the abundances of the fecal Family XIII AD3011 group and *Bacteroides*, both of which were elevated in cows with milk SCC exceeding 50 × 10^4^ cells/mL [154]. Another study reported increased ruminal *Moryella* abundance and decreased fecal *Aeriscardovia*, *Lactococcus*, and *Bacillus* levels in mastitis cows. These findings suggest a potential association between ruminal and fecal microbiota during mastitis. However, whether fecal microorganisms can accurately reflect changes in the ruminal microbiota, particularly in response to probiotic or dietary interventions, requires further investigation. Additionally, more research is needed to validate the effectiveness of targeting the ruminal microbiota through prebiotics or dietary components in mitigating ruminal dysbiosis-induced mastitis.

## Conclusions and perspectives

Mastitis is a major concern for the dairy industry and milk quality, as it is not only triggered by exogenous pathogen invasion but also associated with ruminal dysbiosis. Ruminal dysbiosis enhances the release of LPS and MDP, leading to increased inflammatory responses in the rumen and compromised gastrointestinal barrier integrity. Elevated ruminal permeability facilitates the translocation of LPS into the bloodstream, which impairs the activity of host anti-inflammatory ALP via Neu, thereby activating the TLR4-NF-κB/NLRP3 signaling pathways and inducing systemic inflammation. Disruption of gastrointestinal barrier function also promotes the proliferation and translocation of pathobionts such as *Stenotrophomonas* into the mammary glands. Proinflammatory cytokines, bacterial components (e.g., LPS and MDP), and pathobionts can compromise the blood-milk barrier, resulting in enhanced recruitment of immune cells and the development of mastitis. Additionally, ruminal dysbiosis increases the production of harmful metabolites such as sialic acid and succinate, which enhance the virulence of pathobionts and exacerbate mastitis by elevating LPS and MDP levels. Conversely, reductions in beneficial metabolites, such as AhR ligands and secondary bile acids, further contribute to the progression of mastitis. Although accumulating evidence supports the involvement of ruminal dysbiosis in mastitis pathogenesis, several significant limitations remain. First, alterations in the ruminal microbiota during mastitis are inconsistent across different experimental models, and studies on the roles of ruminal viruses, fungi, and protozoa are limited. Second, specific cellular transcriptional profiles in response to ruminal dysbiosis-induced mastitis remain poorly understood. Finally, RMT studies in dairy cows are essential for confirming the role of ruminal dysbiosis in dairy mastitis, and large-scale clinical trials are needed to validate the targeted regulation of the ruminal microbiota for mastitis prevention and treatment.

## Data Availability

Not applicable.

## Abbreviations

5-HIAA: 5-Hydroxyindole acetic acid
AhR: Aryl hydrocarbon receptor
ALP: Alkaline phosphatase
*B. fibrisolvens*: *Butyrivibrio fibrisolvens*
BCAAs: Branched-chain amino acids
BHBA: β-Hydroxybutyrate
CA: Cholic acid
CAZymes: Carbohydrate-active enzymes
DCA: Deoxycholic acid
DMI: Dry matter intake
*E. coli*: *Escherichia coli*
*F. succinogenes*: *Fibrobacter succinogenes*
FMT: Fecal microbiota transplantation
*L. reuteri*: *Lactobacillus reuteri*
LPS: Lipopolysaccharide
*M. elsdenii*: *Megasphaera elsdenii*
MDP: Muramyl dipeptide
NEB: Negative energy balance
Neu: Neuraminidase
Neu5Ac: N-acetylneuraminic acid
Neu5Gc: N-glycolylneuraminic acid
NOD2: Nucleotide-binding oligomerization domain-containing protein 2
*R. flavefaciens*: *Ruminococcus flavefaciens*
RMT: Ruminal microbiota transplantation
ROS: Reactive oxygen species
*S. aureus*: *Staphylococcus aureus*
*S. bovis*: *Streptococcus bovis*
S. ruminantium: *Selenomonas ruminantium*
SARA: Subacute ruminal acidosis
SCC: Somatic cell count
SCFAs: Short-chain fatty acids
TJs: Tight junctions
TLR4: Toll-like receptor 4
VFA: Volatile fatty acid
